# 3,3′-Dimethyl-1,1′-(methyl­enedi-*p*-phenyl­ene)diimidazolium bis­(hexa­fluoro­phosphate)

**DOI:** 10.1107/S1600536810029727

**Published:** 2010-08-04

**Authors:** Kun Huang, Ping Zhang, Da-Bin Qin

**Affiliations:** a School of Chemistry and Chemical Engineering, China West Normal University, Nanchong 637002, People’s Republic of China

## Abstract

The title *N*-heterocyclic carbene compound, C_21_H_22_N_4_^2+^·2PF_6_^−^, crystallizes as an inversion twin. There are two independent *N*-heterocyclic carbene dications (*A* and *B*) and four independent hexa­fluoro­phosphate anions in the asymmetric unit. The cations are L-shaped with the benzene rings being inclined to one another by 88.82 (16)° in cation *A* and 87.03 (16)° in cation *B*. The imidazole rings make dihedral angles of 35.7 (2) and 32.83 (18)° with the attached benzene rings in cation *A*, and 30.14 (19) and 31.96 (18)° in cation *B*. In the crystal, the cations are linked *via* C—H⋯F hydrogen bonds, forming a three-dimensional network. π–π inter­actions involving the benzene and imidazole rings [centroid–centroid distances = 3.602 (2) and 3.723 (2) Å] and C—H⋯π inter­actions are also present.

## Related literature

For details of the first free carbenes isolated, see: Arduengo *et al.* (1991 ▶). For the role of *N*-heterocyclic carbene ligands in organometallic chemistry, see: Lin *et al.* (2009 ▶). For the synthesis of the title compound, see: Austin *et al.* (1981 ▶); Wei *et al.* (2008 ▶). For a related structure, see: Pinto *et al.* (2009 ▶). For standard bond lengths, see: Allen *et al.* (1987 ▶).
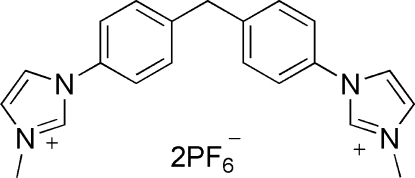


## Experimental

### 

#### Crystal data

C_21_H_22_N_4_^2+^·2PF_6_^−^
*M* *_r_* = 620.37
Orthorhombic, 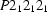

*a* = 11.5645 (19) Å
*b* = 13.690 (2) Å
*c* = 31.188 (5) Å
*V* = 4937.6 (13) Å^3^
*Z* = 8
Mo *K*α radiation
μ = 0.29 mm^−1^
*T* = 113 K
0.20 × 0.18 × 0.12 mm

#### Data collection

Rigaku Saturn CCD area-detector diffractometer
Absorption correction: multi-scan (*CrystalClear*; Rigaku/MSC, 2004 ▶ *T*_min_ = 0.945, *T*_max_ = 0.966
42536 measured reflections
11808 independent reflections
10632 reflections with *I* > 2σ(*I*)
*R*_int_ = 0.064

#### Refinement

*R*[*F* ^2^ > 2σ(*F* ^2^)] = 0.060
*wR*(*F* ^2^) = 0.142
*S* = 1.08
11808 reflections
708 parameters
H-atom parameters constrained
Δρ_max_ = 0.49 e Å^−3^
Δρ_min_ = −0.53 e Å^−3^
Absolute structure: Flack (1983 ▶), 5268 Friedel pairs
Flack parameter: 0.50 (9)

### 

Data collection: *CrystalClear* (Rigaku/MSC, 2004 ▶); cell refinement: *CrystalClear*; data reduction: *CrystalClear*; program(s) used to solve structure: *SHELXS97* (Sheldrick, 2008 ▶); program(s) used to refine structure: *SHELXL97* (Sheldrick, 2008 ▶); molecular graphics: *SHELXTL* (Sheldrick, 2008 ▶); software used to prepare material for publication: *CrystalStructure* (Rigaku/MSC, 2004 ▶).

## Supplementary Material

Crystal structure: contains datablocks global, I. DOI: 10.1107/S1600536810029727/su2188sup1.cif

Structure factors: contains datablocks I. DOI: 10.1107/S1600536810029727/su2188Isup2.hkl

Additional supplementary materials:  crystallographic information; 3D view; checkCIF report

## Figures and Tables

**Table 1:** Hydrogen-bond geometry (Å, °) *Cg*3 and *Cg*7 are the centroids of the C5–C10 and C26–C31 rings, respectively.

*D*—H⋯*A*	*D*—H	H⋯*A*	*D*⋯*A*	*D*—H⋯*A*
C1—H1*A*⋯F11^i^	0.98	2.50	3.345 (5)	144
C2—H2⋯F9^i^	0.95	2.32	3.243 (5)	163
C3—H3⋯F8^ii^	0.95	2.38	3.322 (5)	170
C4—H4⋯F19^ii^	0.95	2.50	3.369 (5)	152
C11—H11*B*⋯F6	0.99	2.40	3.271 (4)	146
C16—H16⋯F10	0.95	2.43	3.098 (5)	128
C18—H18⋯F12	0.95	2.42	3.315 (4)	156
C20—H20⋯F20^iii^	0.95	2.38	3.284 (4)	160
C20—H20⋯F21^iii^	0.95	2.55	3.123 (5)	119
C21—H21*B*⋯F4^iv^	0.98	2.42	3.269 (5)	144
C22—H22*C*⋯F1^v^	0.98	2.35	3.074 (5)	130
C22—H22*C*⋯F17^v^	0.98	2.38	3.239 (5)	146
C23—H23⋯F13^v^	0.95	2.40	3.330 (5)	167
C24—H24⋯F14^iv^	0.95	2.48	3.086 (4)	122
C24—H24⋯F17^iv^	0.95	2.49	3.374 (5)	155
C30—H30⋯F4^iv^	0.95	2.52	3.024 (4)	114
C34—H34⋯F24^i^	0.95	2.43	3.289 (4)	150
C38—H38⋯F23^iii^	0.95	2.53	3.262 (5)	134
C39—H39⋯F16^vi^	0.95	2.45	3.289 (4)	147
C41—H41⋯F2^iii^	0.95	2.39	3.229 (4)	147
C42—H42*B*⋯F22^vi^	0.98	2.35	3.217 (4)	147
C1—H1*B*⋯*Cg*7^vii^	0.98	2.55	3.387 (5)	144
C25—H25⋯*Cg*3^v^	0.95	2.96	3.805 (4)	149

Symmetry codes: (i) 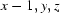; (ii) 
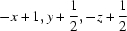
; (iii) 
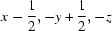
; (iv) 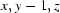; (v) 
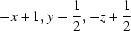
; (vi) 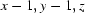; (vii) 
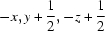
.

**Fig. 1.**
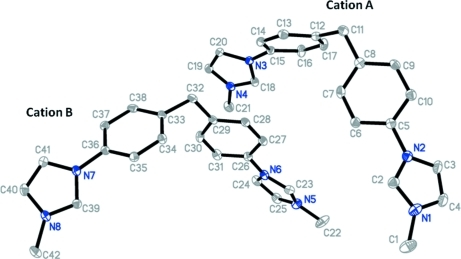
A view of the molecular structures of the two independent cations (A and B) of the title compound. The displacement ellipsoids are drawn at 30% probability level. C-bound H atoms and hexafluorophosphate anions have been omitted for clarity.

**Fig. 2.**
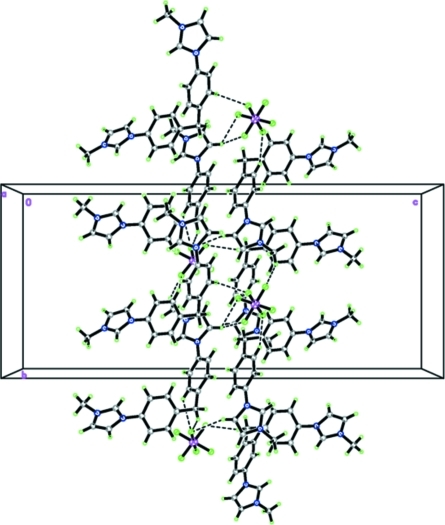
Crystal packing of the title compound, view along the a axis, showing the cations and anions linked via C-H···F interactions (dashed lines), so forming a three-dimensional network.

## supplementary crystallographic information

### Comment

Since the first free carbene was isolated by (Arduengo *et al.*, 1991), N-heterocyclic carbene (NHC) ligands have gradually take up important roles in organometallic chemistry (Lin *et al.*, 2009). Herein we report on the crystal structure of the title N-heterocyclic carbene compound.

The molecular structures of the two independent N-heterocyclic carbene dications (cation A and cation B) are illustrated in Fig. 1. The bond lengths (Allen *et al.*, 1987) and angles are within normal ranges. In the cations the benzene rings make dihedral angles of 88.82 (16) ° in cation A and 87.03 (16) ° in cation B. The imidazole rings are twisted with respect to the attached benzene rings being inclined to one another by 35.7 (2)° and 32.83 (18)° in cation A and 30.14 (19)° and 31.96 (18)° in cation B.

In the crystal there is an extensive arrangement of C—H···F hydrogen bonds, which not only stabilizes the molecular structure but also link the cations (Table 1 and Fig. 2) to form a three-dimensional network. There are also π–π interactions involving rings N3/C18/N4/C19/C20 and C26—C31 [symmetry code: x, y, z], with the ring centroids being separated by 3.602 (2) Å, and rings C5-C10 and N7/C39/N8/C40/C41 [symmetry code: x, y+1, z] with ring centroids separated by 3.723 (2) Å. In addition C—H···π interactions, involving the imidazole and benzene rings, are also present (Table 1).

### Experimental

The title compound was prepared according to the reported procedures (Austin *et al.*, 1981; Wei *et al.*, 2008). Colourless single crystals suitable for X-ray diffraction were obtained by recrystallization from acetonitrile and ethyl ether (v:v = 1:1).

### Refinement

The structure was refined as an inversion twin with the final refined Flack factor being 0.50 (9). H-atoms were placed in calculated positions with C—H = 0.95–0.99 Å, and refined in the riding mode with *U*_iso_(H) = 1.2*U*_eq_(C).

### Crystal data

**Table:**

C_21_H_22_N_4_^2+^·2PF_6_^−^	*F*(000) = 2512
*M**_r_* = 620.37	*D*_x_ = 1.669 Mg m^−^^3^
Orthorhombic, *P*2_1_2_1_2_1_	Mo *K*α radiation, λ = 0.71073 Å
Hall symbol: P 2ac 2ab	Cell parameters from 17727 reflections
*a* = 11.5645 (19) Å	θ = 1.6–27.9°
*b* = 13.690 (2) Å	µ = 0.29 mm^−^^1^
*c* = 31.188 (5) Å	*T* = 113 K
*V* = 4937.6 (13) Å^3^	Prism, colourless
*Z* = 8	0.20 × 0.18 × 0.12 mm

### Data collection

**Table:**

Rigaku Saturn CCD area-detector diffractometer	11808 independent reflections
Radiation source: fine-focus sealed tube	10632 reflections with *I* > 2σ(*I*)
multilayer	*R*_int_ = 0.064
Detector resolution: 14.63 pixels mm^-1^	θ_max_ = 27.9°, θ_min_ = 1.6°
ω and φ scans	*h* = −11→15
Absorption correction: multi-scan (*CrystalClear*; Rigaku/MSC, 2004	*k* = −16→18
*T*_min_ = 0.945, *T*_max_ = 0.966	*l* = −41→38
42536 measured reflections	

### Refinement

**Table:**

Refinement on *F*^2^	Secondary atom site location: difference Fourier map
Least-squares matrix: full	Hydrogen site location: inferred from neighbouring sites
*R*[*F*^2^ > 2σ(*F*^2^)] = 0.060	H-atom parameters constrained
*wR*(*F*^2^) = 0.142	*w* = 1/[σ^2^(*F*_o_^2^) + (0.0571*P*)^2^ + 1.7414*P*] where *P* = (*F*_o_^2^ + 2*F*_c_^2^)/3
*S* = 1.08	(Δ/σ)_max_ < 0.001
11808 reflections	Δρ_max_ = 0.49 e Å^−^^3^
708 parameters	Δρ_min_ = −0.53 e Å^−^^3^
0 restraints	Absolute structure: Flack (1983), 5268 Friedel pairs
Primary atom site location: structure-invariant direct methods	Flack parameter: 0.50 (9)

### Special details

**Table:**

Geometry. Bond distances, angles etc. have been calculated using the rounded fractional coordinates. All su's are estimated from the variances of the (full) variance-covariance matrix. The cell esds are taken into account in the estimation of distances, angles and torsion angles
Refinement. Refinement of F^2^ against ALL reflections. The weighted R-factor wR and goodness of fit S are based on F^2^, conventional R-factors R are based on F, with F set to zero for negative F^2^. The threshold expression of F^2^ > 2sigma(F^2^) is used only for calculating R-factors(gt) etc. and is not relevant to the choice of reflections for refinement. R-factors based on F^2^ are statistically about twice as large as those based on F, and R- factors based on ALL data will be even larger.

### Fractional atomic coordinates and isotropic or equivalent isotropic displacement parameters (Å^2^)

**Table:**

	*x*	*y*	*z*	*U*_iso_*/*U*_eq_	
N1	−0.0473 (3)	0.6747 (3)	0.28054 (10)	0.0401 (10)	
N2	0.0564 (3)	0.7088 (2)	0.22569 (9)	0.0292 (8)	
N3	0.5528 (2)	0.32968 (19)	0.05976 (8)	0.0254 (8)	
N4	0.6505 (2)	0.21221 (19)	0.08809 (9)	0.0285 (8)	
C1	−0.1278 (4)	0.6224 (4)	0.30890 (13)	0.0533 (14)	
C2	−0.0293 (3)	0.6515 (3)	0.23960 (12)	0.0333 (11)	
C3	0.0923 (4)	0.7690 (3)	0.25863 (12)	0.0383 (11)	
C4	0.0270 (4)	0.7479 (3)	0.29267 (12)	0.0406 (11)	
C5	0.1090 (3)	0.7018 (2)	0.18380 (10)	0.0259 (9)	
C6	0.1196 (3)	0.6104 (2)	0.16471 (11)	0.0309 (10)	
C7	0.1750 (3)	0.6042 (2)	0.12554 (11)	0.0333 (10)	
C8	0.2194 (3)	0.6858 (2)	0.10487 (11)	0.0273 (9)	
C9	0.2088 (3)	0.7760 (2)	0.12527 (11)	0.0291 (10)	
C10	0.1522 (3)	0.7850 (3)	0.16437 (11)	0.0323 (10)	
C11	0.2783 (3)	0.6772 (3)	0.06154 (11)	0.0318 (10)	
C12	0.3544 (3)	0.5880 (2)	0.05757 (10)	0.0267 (9)	
C13	0.3195 (3)	0.5064 (2)	0.03432 (10)	0.0310 (10)	
C14	0.3854 (3)	0.4226 (2)	0.03363 (11)	0.0296 (10)	
C15	0.4886 (3)	0.4192 (2)	0.05605 (10)	0.0240 (8)	
C16	0.5287 (3)	0.5010 (2)	0.07769 (12)	0.0328 (10)	
C17	0.4601 (3)	0.5841 (3)	0.07841 (12)	0.0324 (10)	
C18	0.6112 (3)	0.3015 (2)	0.09451 (11)	0.0279 (9)	
C19	0.6158 (3)	0.1818 (2)	0.04820 (11)	0.0314 (10)	
C20	0.5551 (3)	0.2539 (2)	0.03025 (10)	0.0294 (10)	
C21	0.7228 (4)	0.1568 (3)	0.11954 (13)	0.0406 (11)	
N5	0.5227 (3)	0.2547 (2)	0.28123 (9)	0.0338 (9)	
N6	0.4818 (2)	0.21150 (19)	0.21667 (9)	0.0266 (8)	
N7	−0.0206 (2)	−0.17354 (18)	0.05923 (8)	0.0237 (8)	
N8	−0.1168 (3)	−0.29169 (19)	0.08910 (9)	0.0276 (8)	
C22	0.5202 (4)	0.3061 (4)	0.32176 (12)	0.0528 (15)	
C23	0.4504 (3)	0.2705 (3)	0.24893 (10)	0.0287 (10)	
C24	0.5762 (3)	0.1566 (3)	0.22969 (12)	0.0326 (10)	
C25	0.6009 (3)	0.1841 (3)	0.26999 (12)	0.0350 (10)	
C26	0.4238 (3)	0.2047 (2)	0.17611 (10)	0.0259 (9)	
C27	0.3690 (3)	0.2853 (2)	0.15914 (11)	0.0280 (9)	
C28	0.3108 (3)	0.2758 (2)	0.12021 (10)	0.0256 (9)	
C29	0.3095 (3)	0.1870 (2)	0.09819 (10)	0.0250 (9)	
C30	0.3663 (3)	0.1080 (2)	0.11633 (11)	0.0315 (10)	
C31	0.4231 (3)	0.1153 (3)	0.15498 (12)	0.0326 (10)	
C32	0.2481 (3)	0.1767 (2)	0.05534 (11)	0.0283 (10)	
C33	0.1731 (3)	0.0865 (2)	0.05303 (10)	0.0245 (9)	
C34	0.0685 (3)	0.0825 (2)	0.07502 (11)	0.0271 (9)	
C35	0.0022 (3)	−0.0010 (2)	0.07627 (11)	0.0269 (9)	
C36	0.0411 (3)	−0.0836 (2)	0.05510 (10)	0.0229 (8)	
C37	0.1420 (3)	−0.0816 (2)	0.03128 (10)	0.0274 (9)	
C38	0.2076 (3)	0.0044 (2)	0.03039 (11)	0.0298 (10)	
C39	−0.0779 (3)	−0.2017 (2)	0.09465 (10)	0.0249 (9)	
C40	−0.0822 (3)	−0.3235 (2)	0.04900 (11)	0.0307 (10)	
C41	−0.0228 (3)	−0.2503 (2)	0.03027 (10)	0.0276 (9)	
C42	−0.1874 (3)	−0.3460 (2)	0.12033 (13)	0.0372 (11)	
P1	0.61677 (8)	0.88387 (6)	0.06933 (3)	0.0279 (2)	
F1	0.6708 (2)	0.83513 (17)	0.11089 (7)	0.0439 (7)	
F2	0.6730 (2)	0.79921 (17)	0.04033 (7)	0.0499 (8)	
F3	0.5659 (2)	0.93295 (16)	0.02705 (7)	0.0432 (7)	
F4	0.5614 (2)	0.96690 (17)	0.09819 (7)	0.0460 (7)	
F5	0.7322 (2)	0.94752 (19)	0.06358 (8)	0.0515 (8)	
F6	0.5023 (2)	0.82036 (17)	0.07381 (8)	0.0504 (8)	
P2	0.66813 (9)	0.47668 (7)	0.21334 (3)	0.0338 (3)	
F7	0.5352 (2)	0.4707 (2)	0.22527 (11)	0.0721 (12)	
F8	0.7010 (3)	0.4328 (2)	0.25867 (8)	0.0656 (10)	
F9	0.8030 (2)	0.48195 (17)	0.20214 (8)	0.0499 (8)	
F10	0.6398 (3)	0.5190 (2)	0.16774 (9)	0.0785 (11)	
F11	0.6758 (3)	0.58452 (17)	0.23195 (9)	0.0603 (9)	
F12	0.6634 (2)	0.36775 (17)	0.19490 (8)	0.0557 (9)	
P3	0.83051 (8)	0.94635 (7)	0.21464 (3)	0.0294 (3)	
F13	0.7940 (3)	0.8980 (2)	0.25859 (8)	0.0721 (11)	
F14	0.8124 (2)	1.05208 (17)	0.23341 (9)	0.0559 (9)	
F15	0.8668 (3)	0.9915 (2)	0.16996 (8)	0.0726 (10)	
F16	0.8492 (3)	0.84028 (19)	0.19490 (10)	0.0685 (10)	
F17	0.6998 (2)	0.94487 (17)	0.19868 (8)	0.0465 (8)	
F18	0.9615 (2)	0.94825 (18)	0.23005 (9)	0.0502 (8)	
P4	0.93255 (9)	0.37704 (7)	0.07805 (3)	0.0339 (3)	
F19	0.8584 (2)	0.34663 (19)	0.11796 (7)	0.0562 (9)	
F20	0.8867 (2)	0.28285 (16)	0.05298 (7)	0.0530 (8)	
F21	1.0041 (4)	0.4072 (3)	0.03809 (11)	0.135 (2)	
F22	0.9790 (2)	0.47003 (16)	0.10268 (8)	0.0504 (8)	
F23	0.8254 (3)	0.4369 (2)	0.06109 (11)	0.0879 (13)	
F24	1.0361 (3)	0.3159 (2)	0.09699 (15)	0.1033 (16)	
H1A	−0.17950	0.58150	0.29160	0.0800*	
H1B	−0.17370	0.66970	0.32520	0.0800*	
H1C	−0.08400	0.58110	0.32880	0.0800*	
H2	−0.06950	0.60360	0.22340	0.0400*	
H3	0.15210	0.81640	0.25730	0.0460*	
H4	0.03130	0.77780	0.32010	0.0490*	
H6	0.08960	0.55370	0.17830	0.0370*	
H7	0.18290	0.54200	0.11230	0.0400*	
H9	0.24090	0.83250	0.11220	0.0350*	
H10	0.14330	0.84710	0.17750	0.0390*	
H11A	0.21840	0.67500	0.03890	0.0380*	
H11B	0.32600	0.73630	0.05660	0.0380*	
H13	0.24910	0.50850	0.01870	0.0370*	
H14	0.36010	0.36730	0.01780	0.0360*	
H16	0.60160	0.50020	0.09170	0.0390*	
H17	0.48630	0.63990	0.09360	0.0390*	
H18	0.62270	0.33950	0.11970	0.0330*	
H19	0.63210	0.12020	0.03560	0.0380*	
H20	0.52030	0.25340	0.00270	0.0350*	
H21A	0.79890	0.14370	0.10700	0.0610*	
H21B	0.68470	0.09480	0.12650	0.0610*	
H21C	0.73210	0.19560	0.14570	0.0610*	
H22A	0.57690	0.35930	0.32130	0.0790*	
H22B	0.53910	0.26070	0.34500	0.0790*	
H22C	0.44280	0.33320	0.32650	0.0790*	
H23	0.38780	0.31550	0.24870	0.0340*	
H24	0.61590	0.10880	0.21330	0.0390*	
H25	0.66130	0.15920	0.28750	0.0420*	
H27	0.37080	0.34630	0.17370	0.0340*	
H28	0.27140	0.33050	0.10850	0.0310*	
H30	0.36590	0.04710	0.10170	0.0380*	
H31	0.46110	0.06020	0.16700	0.0390*	
H32A	0.19930	0.23510	0.05050	0.0340*	
H32B	0.30650	0.17400	0.03220	0.0340*	
H34	0.04190	0.13920	0.08960	0.0330*	
H35	−0.06910	−0.00200	0.09140	0.0320*	
H37	0.16630	−0.13760	0.01580	0.0330*	
H38	0.27690	0.00640	0.01400	0.0360*	
H39	−0.08880	−0.16310	0.11970	0.0300*	
H40	−0.09760	−0.38580	0.03680	0.0370*	
H41	0.01110	−0.25120	0.00250	0.0330*	
H42A	−0.19200	−0.30900	0.14720	0.0560*	
H42B	−0.15190	−0.40980	0.12590	0.0560*	
H42C	−0.26540	−0.35550	0.10870	0.0560*	

### Atomic displacement parameters (Å^2^)

**Table:**

	*U*^11^	*U*^22^	*U*^33^	*U*^12^	*U*^13^	*U*^23^
N1	0.0387 (18)	0.0517 (19)	0.0299 (16)	0.0071 (16)	0.0049 (14)	−0.0031 (14)
N2	0.0317 (15)	0.0268 (14)	0.0290 (14)	0.0056 (12)	−0.0030 (12)	0.0008 (11)
N3	0.0267 (14)	0.0252 (13)	0.0243 (13)	−0.0063 (11)	0.0018 (11)	−0.0015 (11)
N4	0.0267 (14)	0.0260 (13)	0.0327 (15)	−0.0042 (12)	0.0036 (12)	0.0027 (11)
C1	0.037 (2)	0.081 (3)	0.042 (2)	0.004 (2)	0.0100 (19)	−0.005 (2)
C2	0.0322 (19)	0.0355 (19)	0.0321 (18)	0.0028 (15)	0.0038 (15)	−0.0028 (15)
C3	0.051 (2)	0.0305 (18)	0.0335 (18)	0.0037 (17)	−0.0063 (18)	−0.0087 (15)
C4	0.052 (2)	0.0357 (19)	0.034 (2)	0.0068 (18)	−0.0059 (18)	−0.0081 (16)
C5	0.0233 (15)	0.0279 (16)	0.0264 (16)	0.0013 (13)	−0.0064 (13)	0.0003 (13)
C6	0.0323 (18)	0.0275 (16)	0.0328 (18)	−0.0049 (15)	0.0035 (15)	−0.0030 (14)
C7	0.0334 (18)	0.0265 (16)	0.0400 (19)	−0.0024 (15)	0.0042 (16)	−0.0084 (14)
C8	0.0189 (14)	0.0313 (17)	0.0316 (17)	0.0000 (13)	−0.0040 (13)	0.0008 (14)
C9	0.0278 (16)	0.0271 (16)	0.0323 (18)	−0.0016 (14)	−0.0061 (14)	0.0054 (13)
C10	0.037 (2)	0.0276 (16)	0.0322 (18)	0.0003 (15)	−0.0059 (15)	−0.0002 (14)
C11	0.0296 (17)	0.0332 (18)	0.0326 (18)	0.0020 (14)	−0.0005 (15)	0.0044 (14)
C12	0.0261 (16)	0.0307 (16)	0.0234 (15)	−0.0005 (13)	0.0022 (13)	0.0010 (12)
C13	0.0295 (17)	0.0360 (18)	0.0275 (17)	−0.0035 (15)	−0.0078 (15)	0.0045 (14)
C14	0.0359 (18)	0.0278 (16)	0.0251 (16)	−0.0052 (14)	−0.0060 (15)	−0.0001 (13)
C15	0.0209 (14)	0.0272 (15)	0.0240 (15)	−0.0014 (12)	0.0018 (12)	−0.0010 (12)
C16	0.0232 (16)	0.0313 (18)	0.044 (2)	−0.0024 (14)	−0.0066 (15)	−0.0101 (15)
C17	0.0256 (17)	0.0305 (17)	0.041 (2)	0.0017 (14)	−0.0055 (15)	−0.0092 (15)
C18	0.0239 (16)	0.0267 (16)	0.0330 (17)	−0.0037 (14)	0.0000 (14)	−0.0018 (13)
C19	0.0352 (18)	0.0268 (16)	0.0322 (18)	−0.0050 (15)	0.0065 (15)	−0.0046 (14)
C20	0.0380 (19)	0.0280 (16)	0.0222 (15)	−0.0055 (15)	0.0032 (14)	−0.0032 (13)
C21	0.038 (2)	0.0358 (19)	0.048 (2)	−0.0050 (17)	−0.0032 (18)	0.0009 (17)
N5	0.0298 (15)	0.0404 (16)	0.0311 (15)	0.0025 (13)	−0.0066 (13)	0.0010 (13)
N6	0.0216 (13)	0.0226 (13)	0.0357 (15)	0.0023 (11)	−0.0047 (12)	0.0011 (12)
N7	0.0234 (13)	0.0201 (12)	0.0276 (14)	0.0002 (10)	−0.0012 (11)	−0.0002 (10)
N8	0.0278 (14)	0.0200 (12)	0.0350 (15)	−0.0027 (11)	−0.0009 (12)	0.0013 (11)
C22	0.053 (3)	0.081 (3)	0.0245 (18)	0.030 (3)	−0.0045 (18)	−0.008 (2)
C23	0.0208 (15)	0.0330 (18)	0.0324 (18)	0.0016 (14)	−0.0024 (13)	0.0061 (14)
C24	0.0242 (17)	0.0306 (17)	0.043 (2)	0.0036 (14)	−0.0054 (15)	0.0038 (15)
C25	0.0282 (17)	0.0308 (17)	0.046 (2)	0.0071 (15)	−0.0043 (16)	0.0059 (15)
C26	0.0196 (14)	0.0282 (16)	0.0298 (16)	−0.0004 (13)	0.0005 (13)	−0.0001 (13)
C27	0.0286 (16)	0.0208 (14)	0.0347 (18)	0.0020 (13)	0.0013 (14)	0.0012 (13)
C28	0.0228 (15)	0.0221 (14)	0.0318 (17)	−0.0005 (12)	0.0022 (13)	0.0031 (13)
C29	0.0216 (15)	0.0238 (15)	0.0296 (16)	−0.0029 (12)	0.0025 (13)	0.0023 (12)
C30	0.0273 (16)	0.0273 (16)	0.0400 (19)	0.0033 (14)	−0.0035 (15)	−0.0070 (14)
C31	0.0254 (16)	0.0263 (16)	0.046 (2)	0.0038 (14)	0.0003 (15)	0.0003 (15)
C32	0.0289 (17)	0.0275 (16)	0.0285 (17)	−0.0055 (14)	0.0039 (13)	0.0043 (13)
C33	0.0244 (15)	0.0254 (15)	0.0237 (15)	−0.0035 (13)	−0.0014 (13)	0.0028 (12)
C34	0.0226 (15)	0.0246 (15)	0.0342 (17)	0.0015 (13)	0.0024 (14)	−0.0042 (13)
C35	0.0224 (15)	0.0280 (16)	0.0303 (17)	−0.0010 (13)	0.0041 (14)	−0.0016 (13)
C36	0.0230 (15)	0.0204 (14)	0.0253 (15)	−0.0004 (12)	−0.0021 (12)	0.0012 (12)
C37	0.0324 (18)	0.0252 (15)	0.0246 (16)	0.0021 (13)	0.0054 (14)	−0.0028 (13)
C38	0.0283 (16)	0.0330 (17)	0.0281 (17)	−0.0017 (14)	0.0109 (14)	0.0025 (14)
C39	0.0263 (16)	0.0230 (15)	0.0255 (16)	0.0005 (13)	−0.0001 (13)	0.0009 (12)
C40	0.0353 (18)	0.0208 (15)	0.0361 (18)	0.0002 (14)	−0.0075 (15)	−0.0026 (13)
C41	0.0281 (17)	0.0278 (15)	0.0268 (16)	0.0020 (13)	−0.0003 (13)	−0.0036 (13)
C42	0.036 (2)	0.0256 (16)	0.050 (2)	−0.0040 (15)	0.0042 (18)	0.0070 (16)
P1	0.0262 (4)	0.0264 (4)	0.0311 (4)	0.0022 (3)	−0.0024 (4)	−0.0035 (3)
F1	0.0430 (13)	0.0567 (14)	0.0320 (11)	0.0184 (12)	−0.0023 (10)	0.0039 (10)
F2	0.0575 (15)	0.0510 (13)	0.0412 (12)	0.0228 (13)	−0.0112 (12)	−0.0173 (10)
F3	0.0463 (13)	0.0426 (12)	0.0408 (12)	0.0060 (11)	−0.0069 (11)	0.0115 (10)
F4	0.0437 (13)	0.0427 (12)	0.0515 (13)	0.0132 (11)	−0.0026 (11)	−0.0172 (10)
F5	0.0348 (12)	0.0648 (16)	0.0550 (15)	−0.0143 (12)	−0.0005 (11)	0.0035 (13)
F6	0.0426 (13)	0.0490 (14)	0.0597 (15)	−0.0191 (12)	−0.0013 (12)	0.0055 (12)
P2	0.0397 (5)	0.0299 (4)	0.0318 (5)	−0.0024 (4)	−0.0004 (4)	0.0008 (4)
F7	0.0439 (15)	0.0474 (15)	0.125 (3)	0.0012 (13)	0.0260 (17)	0.0104 (16)
F8	0.099 (2)	0.0623 (17)	0.0356 (13)	0.0115 (17)	−0.0007 (15)	0.0086 (12)
F9	0.0412 (13)	0.0437 (13)	0.0649 (16)	−0.0062 (11)	0.0007 (12)	−0.0083 (12)
F10	0.085 (2)	0.097 (2)	0.0535 (16)	−0.0338 (19)	−0.0342 (16)	0.0383 (16)
F11	0.0624 (17)	0.0314 (12)	0.0872 (19)	0.0051 (13)	0.0032 (16)	−0.0113 (12)
F12	0.0585 (16)	0.0430 (13)	0.0657 (16)	−0.0132 (13)	0.0097 (14)	−0.0210 (12)
P3	0.0247 (4)	0.0306 (4)	0.0328 (5)	0.0004 (4)	−0.0003 (4)	−0.0032 (4)
F13	0.073 (2)	0.099 (2)	0.0444 (14)	−0.0320 (18)	0.0006 (14)	0.0211 (15)
F14	0.0338 (12)	0.0390 (12)	0.095 (2)	0.0037 (11)	−0.0180 (13)	−0.0300 (13)
F15	0.0578 (18)	0.111 (2)	0.0489 (15)	−0.0018 (18)	0.0069 (14)	0.0258 (16)
F16	0.0642 (19)	0.0473 (15)	0.094 (2)	0.0192 (14)	−0.0251 (16)	−0.0309 (14)
F17	0.0307 (11)	0.0501 (14)	0.0586 (15)	−0.0030 (10)	−0.0098 (11)	−0.0166 (11)
F18	0.0314 (12)	0.0417 (13)	0.0774 (18)	0.0047 (10)	−0.0163 (12)	0.0051 (12)
P4	0.0371 (5)	0.0230 (4)	0.0416 (5)	−0.0049 (4)	0.0082 (4)	−0.0034 (4)
F19	0.0601 (17)	0.0684 (16)	0.0401 (13)	−0.0261 (14)	0.0056 (12)	−0.0041 (12)
F20	0.0705 (17)	0.0399 (12)	0.0487 (13)	−0.0230 (13)	0.0196 (13)	−0.0164 (10)
F21	0.211 (5)	0.102 (3)	0.093 (2)	−0.112 (3)	0.104 (3)	−0.052 (2)
F22	0.0496 (14)	0.0349 (12)	0.0668 (16)	−0.0081 (11)	0.0000 (13)	−0.0161 (11)
F23	0.115 (3)	0.0518 (16)	0.097 (2)	0.0150 (18)	−0.068 (2)	0.0036 (16)
F24	0.0421 (17)	0.0489 (17)	0.219 (4)	0.0170 (14)	−0.029 (2)	−0.032 (2)

### Geometric parameters (Å, °)

**Table:**

P1—F2	1.608 (2)	C16—C17	1.387 (5)
P1—F3	1.593 (2)	C19—C20	1.334 (4)
P1—F4	1.585 (2)	C1—H1C	0.9800
P1—F5	1.604 (3)	C1—H1B	0.9800
P1—F6	1.590 (2)	C1—H1A	0.9800
P1—F1	1.586 (2)	C2—H2	0.9500
P2—F7	1.584 (3)	C3—H3	0.9500
P2—F10	1.570 (3)	C4—H4	0.9500
P2—F11	1.589 (3)	C6—H6	0.9500
P2—F12	1.599 (3)	C7—H7	0.9500
P2—F8	1.582 (3)	C9—H9	0.9500
P2—F9	1.600 (3)	C10—H10	0.9500
P3—F15	1.581 (3)	C11—H11A	0.9900
P3—F13	1.580 (3)	C11—H11B	0.9900
P3—F14	1.575 (3)	C13—H13	0.9500
P3—F17	1.592 (3)	C14—H14	0.9500
P3—F18	1.590 (3)	C16—H16	0.9500
P3—F16	1.592 (3)	C17—H17	0.9500
P4—F19	1.568 (2)	C18—H18	0.9500
P4—F21	1.552 (4)	C19—H19	0.9500
P4—F22	1.581 (2)	C20—H20	0.9500
P4—F20	1.599 (2)	C21—H21C	0.9800
P4—F24	1.576 (4)	C21—H21A	0.9800
P4—F23	1.577 (3)	C21—H21B	0.9800
N1—C2	1.332 (5)	C24—C25	1.343 (5)
N1—C4	1.373 (6)	C26—C31	1.390 (5)
N1—C1	1.470 (6)	C26—C27	1.378 (4)
N2—C5	1.444 (4)	C27—C28	1.394 (5)
N2—C2	1.336 (5)	C28—C29	1.396 (4)
N2—C3	1.381 (5)	C29—C32	1.520 (5)
N3—C15	1.438 (4)	C29—C30	1.386 (4)
N3—C18	1.334 (4)	C30—C31	1.376 (5)
N3—C20	1.387 (4)	C32—C33	1.511 (4)
N4—C18	1.319 (4)	C33—C34	1.392 (5)
N4—C19	1.372 (4)	C33—C38	1.386 (4)
N4—C21	1.496 (5)	C34—C35	1.377 (4)
N5—C22	1.447 (5)	C35—C36	1.385 (4)
N5—C23	1.327 (5)	C36—C37	1.384 (5)
N5—C25	1.369 (5)	C37—C38	1.401 (4)
N6—C23	1.340 (4)	C40—C41	1.348 (4)
N6—C24	1.386 (4)	C22—H22B	0.9800
N6—C26	1.435 (4)	C22—H22C	0.9800
N7—C41	1.386 (4)	C22—H22A	0.9800
N7—C39	1.345 (4)	C23—H23	0.9500
N7—C36	1.429 (4)	C24—H24	0.9500
N8—C40	1.383 (4)	C25—H25	0.9500
N8—C39	1.323 (4)	C27—H27	0.9500
N8—C42	1.472 (5)	C28—H28	0.9500
C3—C4	1.335 (6)	C30—H30	0.9500
C5—C10	1.384 (5)	C31—H31	0.9500
C5—C6	1.391 (4)	C32—H32A	0.9900
C6—C7	1.382 (5)	C32—H32B	0.9900
C7—C8	1.388 (4)	C34—H34	0.9500
C8—C11	1.518 (5)	C35—H35	0.9500
C8—C9	1.395 (4)	C37—H37	0.9500
C9—C10	1.390 (5)	C38—H38	0.9500
C11—C12	1.510 (5)	C39—H39	0.9500
C12—C13	1.392 (4)	C40—H40	0.9500
C12—C17	1.386 (5)	C41—H41	0.9500
C13—C14	1.378 (4)	C42—H42C	0.9800
C14—C15	1.384 (5)	C42—H42A	0.9800
C15—C16	1.387 (4)	C42—H42B	0.9800
			
F2—P1—F3	89.30 (12)	H1A—C1—H1B	109.00
F2—P1—F4	179.63 (13)	H1B—C1—H1C	109.00
F2—P1—F5	89.55 (13)	N1—C1—H1C	110.00
F2—P1—F6	89.54 (13)	N2—C2—H2	127.00
F3—P1—F4	91.05 (12)	N1—C2—H2	127.00
F3—P1—F5	89.18 (13)	C4—C3—H3	127.00
F3—P1—F6	89.77 (13)	N2—C3—H3	126.00
F4—P1—F5	90.58 (13)	C3—C4—H4	126.00
F4—P1—F6	90.34 (13)	N1—C4—H4	127.00
F5—P1—F6	178.61 (14)	C7—C6—H6	121.00
F1—P1—F2	89.84 (12)	C5—C6—H6	121.00
F1—P1—F3	178.46 (13)	C8—C7—H7	119.00
F1—P1—F4	89.82 (12)	C6—C7—H7	119.00
F1—P1—F5	89.55 (13)	C10—C9—H9	119.00
F1—P1—F6	91.50 (13)	C8—C9—H9	119.00
F8—P2—F11	90.74 (15)	C9—C10—H10	121.00
F8—P2—F12	88.60 (14)	C5—C10—H10	121.00
F9—P2—F10	89.37 (16)	C8—C11—H11B	109.00
F9—P2—F11	89.05 (16)	C12—C11—H11A	109.00
F9—P2—F12	89.82 (13)	H11A—C11—H11B	108.00
F10—P2—F11	89.98 (15)	C8—C11—H11A	109.00
F10—P2—F12	90.64 (14)	C12—C11—H11B	109.00
F11—P2—F12	178.71 (17)	C14—C13—H13	119.00
F8—P2—F9	88.74 (16)	C12—C13—H13	119.00
F8—P2—F10	177.97 (19)	C15—C14—H14	120.00
F7—P2—F8	90.21 (18)	C13—C14—H14	120.00
F7—P2—F9	178.95 (16)	C17—C16—H16	121.00
F7—P2—F10	91.68 (18)	C15—C16—H16	121.00
F7—P2—F11	90.94 (17)	C12—C17—H17	119.00
F7—P2—F12	90.18 (14)	C16—C17—H17	119.00
F13—P3—F14	91.55 (15)	N4—C18—H18	126.00
F13—P3—F15	178.21 (15)	N3—C18—H18	126.00
F14—P3—F18	89.96 (13)	C20—C19—H19	126.00
F15—P3—F16	88.84 (16)	N4—C19—H19	126.00
F15—P3—F17	88.94 (16)	C19—C20—H20	127.00
F15—P3—F18	90.41 (16)	N3—C20—H20	127.00
F16—P3—F17	89.79 (16)	H21B—C21—H21C	110.00
F16—P3—F18	90.14 (16)	N4—C21—H21A	109.00
F17—P3—F18	179.34 (15)	H21A—C21—H21B	109.00
F14—P3—F17	90.10 (13)	H21A—C21—H21C	109.00
F13—P3—F18	89.96 (16)	N4—C21—H21B	109.00
F13—P3—F16	89.41 (16)	N4—C21—H21C	109.00
F13—P3—F17	90.70 (16)	N5—C23—N6	107.5 (3)
F14—P3—F15	90.21 (15)	N6—C24—C25	106.8 (3)
F14—P3—F16	179.04 (16)	N5—C25—C24	107.3 (3)
F19—P4—F21	179.05 (19)	N6—C26—C27	120.1 (3)
F19—P4—F20	89.58 (13)	N6—C26—C31	118.5 (3)
F21—P4—F22	89.70 (18)	C27—C26—C31	121.4 (3)
F21—P4—F23	90.7 (2)	C26—C27—C28	118.8 (3)
F21—P4—F24	92.1 (2)	C27—C28—C29	121.0 (3)
F22—P4—F23	90.66 (15)	C28—C29—C30	118.3 (3)
F22—P4—F24	89.28 (16)	C28—C29—C32	121.2 (3)
F23—P4—F24	177.2 (2)	C30—C29—C32	120.5 (3)
F20—P4—F22	179.50 (14)	C29—C30—C31	121.8 (3)
F20—P4—F23	89.68 (15)	C26—C31—C30	118.8 (3)
F20—P4—F24	90.40 (16)	C29—C32—C33	112.7 (3)
F19—P4—F22	90.80 (14)	C32—C33—C34	120.5 (3)
F19—P4—F23	88.54 (16)	C32—C33—C38	121.5 (3)
F19—P4—F24	88.68 (18)	C34—C33—C38	118.0 (3)
F20—P4—F21	89.92 (18)	C33—C34—C35	122.1 (3)
C1—N1—C2	124.0 (4)	C34—C35—C36	118.9 (3)
C1—N1—C4	125.9 (3)	C35—C36—C37	120.9 (3)
C2—N1—C4	109.9 (3)	N7—C36—C37	119.1 (3)
C3—N2—C5	125.9 (3)	N7—C36—C35	119.9 (3)
C2—N2—C5	124.6 (3)	C36—C37—C38	119.0 (3)
C2—N2—C3	109.4 (3)	C33—C38—C37	121.0 (3)
C18—N3—C20	108.2 (2)	N7—C39—N8	109.1 (3)
C15—N3—C20	126.5 (3)	N8—C40—C41	107.8 (3)
C15—N3—C18	125.0 (3)	N7—C41—C40	106.9 (3)
C18—N4—C19	108.5 (3)	N5—C22—H22B	109.00
C18—N4—C21	124.3 (3)	N5—C22—H22C	110.00
C19—N4—C21	127.2 (3)	H22A—C22—H22B	109.00
C22—N5—C23	124.8 (3)	N5—C22—H22A	110.00
C22—N5—C25	125.5 (3)	H22A—C22—H22C	109.00
C23—N5—C25	109.7 (3)	H22B—C22—H22C	109.00
C24—N6—C26	126.2 (3)	N5—C23—H23	126.00
C23—N6—C24	108.7 (3)	N6—C23—H23	126.00
C23—N6—C26	125.1 (3)	N6—C24—H24	127.00
C36—N7—C39	124.6 (3)	C25—C24—H24	126.00
C36—N7—C41	127.1 (3)	N5—C25—H25	126.00
C39—N7—C41	108.0 (2)	C24—C25—H25	126.00
C39—N8—C40	108.2 (3)	C26—C27—H27	121.00
C40—N8—C42	126.8 (3)	C28—C27—H27	121.00
C39—N8—C42	124.9 (3)	C27—C28—H28	120.00
N1—C2—N2	106.7 (3)	C29—C28—H28	119.00
N2—C3—C4	107.0 (4)	C31—C30—H30	119.00
N1—C4—C3	107.0 (3)	C29—C30—H30	119.00
N2—C5—C6	118.9 (3)	C26—C31—H31	121.00
C6—C5—C10	121.4 (3)	C30—C31—H31	121.00
N2—C5—C10	119.6 (3)	H32A—C32—H32B	108.00
C5—C6—C7	118.3 (3)	C29—C32—H32B	109.00
C6—C7—C8	122.2 (3)	C33—C32—H32A	109.00
C7—C8—C11	121.1 (3)	C29—C32—H32A	109.00
C7—C8—C9	117.9 (3)	C33—C32—H32B	109.00
C9—C8—C11	121.0 (3)	C35—C34—H34	119.00
C8—C9—C10	121.4 (3)	C33—C34—H34	119.00
C5—C10—C9	118.8 (3)	C34—C35—H35	121.00
C8—C11—C12	113.4 (3)	C36—C35—H35	120.00
C11—C12—C13	121.5 (3)	C36—C37—H37	120.00
C13—C12—C17	118.0 (3)	C38—C37—H37	121.00
C11—C12—C17	120.5 (3)	C37—C38—H38	119.00
C12—C13—C14	121.1 (3)	C33—C38—H38	120.00
C13—C14—C15	119.8 (3)	N7—C39—H39	125.00
N3—C15—C14	121.0 (3)	N8—C39—H39	125.00
N3—C15—C16	118.5 (3)	N8—C40—H40	126.00
C14—C15—C16	120.5 (3)	C41—C40—H40	126.00
C15—C16—C17	118.6 (3)	N7—C41—H41	127.00
C12—C17—C16	121.9 (3)	C40—C41—H41	127.00
N3—C18—N4	108.6 (3)	H42A—C42—H42C	110.00
N4—C19—C20	108.1 (3)	H42B—C42—H42C	109.00
N3—C20—C19	106.6 (3)	H42A—C42—H42B	109.00
H1A—C1—H1C	110.00	N8—C42—H42A	110.00
N1—C1—H1B	109.00	N8—C42—H42B	110.00
N1—C1—H1A	109.00	N8—C42—H42C	109.00
			
C1—N1—C2—N2	−174.4 (4)	N2—C5—C10—C9	176.1 (3)
C4—N1—C2—N2	0.5 (5)	C6—C5—C10—C9	−1.0 (5)
C1—N1—C4—C3	174.1 (4)	C10—C5—C6—C7	0.1 (5)
C2—N1—C4—C3	−0.6 (5)	C5—C6—C7—C8	−0.3 (5)
C3—N2—C2—N1	−0.1 (4)	C6—C7—C8—C9	1.3 (5)
C5—N2—C2—N1	174.9 (3)	C6—C7—C8—C11	−179.1 (3)
C2—N2—C3—C4	−0.3 (5)	C7—C8—C9—C10	−2.2 (5)
C5—N2—C3—C4	−175.2 (3)	C11—C8—C9—C10	178.2 (3)
C2—N2—C5—C6	−33.9 (5)	C7—C8—C11—C12	−39.9 (5)
C2—N2—C5—C10	148.9 (4)	C9—C8—C11—C12	139.7 (3)
C3—N2—C5—C6	140.2 (4)	C8—C9—C10—C5	2.0 (5)
C3—N2—C5—C10	−36.9 (5)	C8—C11—C12—C13	103.9 (4)
C18—N3—C15—C14	−143.1 (3)	C8—C11—C12—C17	−73.9 (4)
C18—N3—C15—C16	33.0 (5)	C11—C12—C13—C14	−174.9 (3)
C20—N3—C15—C14	29.7 (5)	C17—C12—C13—C14	3.0 (5)
C20—N3—C15—C16	−154.3 (3)	C13—C12—C17—C16	−2.1 (5)
C15—N3—C18—N4	174.3 (3)	C11—C12—C17—C16	175.8 (3)
C20—N3—C18—N4	0.4 (4)	C12—C13—C14—C15	−0.6 (5)
C15—N3—C20—C19	−174.0 (3)	C13—C14—C15—C16	−2.8 (5)
C18—N3—C20—C19	−0.3 (4)	C13—C14—C15—N3	173.2 (3)
C19—N4—C18—N3	−0.4 (4)	N3—C15—C16—C17	−172.4 (3)
C21—N4—C18—N3	178.4 (3)	C14—C15—C16—C17	3.7 (5)
C18—N4—C19—C20	0.2 (4)	C15—C16—C17—C12	−1.2 (5)
C21—N4—C19—C20	−178.5 (3)	N4—C19—C20—N3	0.0 (4)
C23—N5—C25—C24	0.4 (4)	N6—C24—C25—N5	−0.1 (4)
C22—N5—C23—N6	178.2 (3)	N6—C26—C27—C28	−178.3 (3)
C25—N5—C23—N6	−0.7 (4)	C31—C26—C27—C28	0.8 (5)
C22—N5—C25—C24	−178.4 (4)	N6—C26—C31—C30	179.2 (3)
C23—N6—C24—C25	−0.3 (4)	C27—C26—C31—C30	0.0 (5)
C26—N6—C24—C25	−178.3 (3)	C26—C27—C28—C29	−1.2 (5)
C23—N6—C26—C27	31.0 (5)	C27—C28—C29—C30	0.9 (5)
C26—N6—C23—N5	178.6 (3)	C27—C28—C29—C32	−178.9 (3)
C24—N6—C23—N5	0.6 (4)	C28—C29—C30—C31	−0.1 (5)
C24—N6—C26—C27	−151.3 (3)	C32—C29—C30—C31	179.7 (3)
C24—N6—C26—C31	29.5 (5)	C28—C29—C32—C33	−133.3 (3)
C23—N6—C26—C31	−148.1 (3)	C30—C29—C32—C33	47.0 (4)
C41—N7—C36—C35	154.4 (3)	C29—C30—C31—C26	−0.4 (5)
C41—N7—C36—C37	−28.3 (5)	C29—C32—C33—C34	74.0 (4)
C39—N7—C36—C35	−32.7 (5)	C29—C32—C33—C38	−103.8 (4)
C39—N7—C36—C37	144.6 (3)	C32—C33—C34—C35	−175.0 (3)
C36—N7—C39—N8	−174.7 (3)	C38—C33—C34—C35	2.8 (5)
C41—N7—C39—N8	−0.7 (4)	C32—C33—C38—C37	174.8 (3)
C36—N7—C41—C40	174.0 (3)	C34—C33—C38—C37	−3.0 (5)
C39—N7—C41—C40	0.2 (4)	C33—C34—C35—C36	0.2 (5)
C39—N8—C40—C41	−0.7 (4)	C34—C35—C36—N7	174.2 (3)
C42—N8—C40—C41	177.7 (3)	C34—C35—C36—C37	−3.1 (5)
C42—N8—C39—N7	−177.6 (3)	N7—C36—C37—C38	−174.5 (3)
C40—N8—C39—N7	0.9 (4)	C35—C36—C37—C38	2.9 (5)
N2—C3—C4—N1	0.6 (5)	C36—C37—C38—C33	0.3 (5)
N2—C5—C6—C7	−177.0 (3)	N8—C40—C41—N7	0.3 (4)

### Hydrogen-bond geometry (Å, °)

**Table:**

Cg3 and Cg7 are the centroids of the C5–C10 and C26–C31 rings, respectively.

**Table:**

*D*—H···*A*	*D*—H	H···*A*	*D*···*A*	*D*—H···*A*
C1—H1A···F11^i^	0.98	2.50	3.345 (5)	144
C2—H2···F9^i^	0.95	2.32	3.243 (5)	163
C3—H3···F8^ii^	0.95	2.38	3.322 (5)	170
C4—H4···F19^ii^	0.95	2.50	3.369 (5)	152
C11—H11B···F6	0.99	2.40	3.271 (4)	146
C16—H16···F10	0.95	2.43	3.098 (5)	128
C18—H18···F12	0.95	2.42	3.315 (4)	156
C20—H20···F20^iii^	0.95	2.38	3.284 (4)	160
C20—H20···F21^iii^	0.95	2.55	3.123 (5)	119
C21—H21B···F4^iv^	0.98	2.42	3.269 (5)	144
C22—H22C···F1^v^	0.98	2.35	3.074 (5)	130
C22—H22C···F17^v^	0.98	2.38	3.239 (5)	146
C23—H23···F13^v^	0.95	2.40	3.330 (5)	167
C24—H24···F14^iv^	0.95	2.48	3.086 (4)	122
C24—H24···F17^iv^	0.95	2.49	3.374 (5)	155
C30—H30···F4^iv^	0.95	2.52	3.024 (4)	114
C34—H34···F24^i^	0.95	2.43	3.289 (4)	150
C38—H38···F23^iii^	0.95	2.53	3.262 (5)	134
C39—H39···F16^vi^	0.95	2.45	3.289 (4)	147
C41—H41···F2^iii^	0.95	2.39	3.229 (4)	147
C42—H42B···F22^vi^	0.98	2.35	3.217 (4)	147
C1—H1B···Cg7^vii^	0.98	2.55	3.387 (5)	144
C25—H25···Cg3^v^	0.95	2.96	3.805 (4)	149

Symmetry codes: (i) *x*−1, *y*, *z*; (ii) −*x*+1, *y*+1/2, −*z*+1/2; (iii) *x*−1/2, −*y*+1/2, −*z*; (iv) *x*, *y*−1, *z*; (v) −*x*+1, *y*−1/2, −*z*+1/2; (vi) *x*−1, *y*−1, *z*; (vii) −*x*, *y*+1/2, −*z*+1/2.
